# Perception of Malaria Chemoprevention Interventions in Infants and Children in Eight Sub-Saharan African Countries: An End User Perspective Study

**DOI:** 10.3390/tropicalmed6020075

**Published:** 2021-05-11

**Authors:** Céline Audibert, André-Marie Tchouatieu

**Affiliations:** Medicines for Malaria Venture, 1215 Geneva, Switzerland; tchouatieua@mmv.org

**Keywords:** malaria chemoprevention, children and infants, implementation, intermittent preventive treatment of infants (IPTi), seasonal malaria chemoprevention (SMC), end user

## Abstract

Preventive chemotherapy interventions have been identified as key tools for malaria prevention and control. Although a large number of publications have reported on the efficacy and safety profile of these interventions, little literature exists on end-user experience. The objective of this study was to provide insights on the perceptions and attitudes towards seasonal malaria chemoprevention (SMC) and intermittent preventive treatment of infants (IPTi) to identify drivers of and barriers to acceptance. A total of 179 in-depth qualitative interviews were conducted with community health workers (CHWs), health center managers, parents of children receiving chemoprevention, and national decision makers across eight countries in sub-Saharan Africa. The transcribed verbatim responses were coded and analyzed using a thematic approach. Findings indicate that, although SMC is largely accepted by end users, coverage remained below 100%. The main causes mentioned were children’s absenteeism, children being sick, parents’ reluctance, and lack of staff. Regarding IPTi, results from participants based in Sierra Leone showed that the intervention was generally accepted and perceived as efficacious. The main challenges were access to water, crushing the tablets, and high staff turnover. SMC and IPTi are perceived as valuable interventions. Our study identified the key elements that need to be considered to facilitate the expansion of these two interventions to different geographies or age groups.

## 1. Introduction

Since 2000, tremendous gains have been made in reducing the burden of malaria thanks to global control efforts. However, since 2016, this progress has plateaued, and malaria remains one of the most devastating infectious diseases, with an estimated 229 million infections and 409,000 deaths in 2019, mainly children under the age of five in sub-Saharan Africa [1]. To reduce the global burden of malaria, the World Health Organization (WHO) published recommendations on preventive chemotherapies, effective vector control, diagnosis, treatment, and elimination [2]. WHO-recommended preventive therapies include intermittent preventive treatment of pregnant women (IPTp), intermittent preventive treatment of infants (IPTi), and seasonal malaria chemoprevention (SMC) [3]. This article focuses on the latter two chemoprevention interventions targeting children and infants.

Seasonal malaria chemoprevention is defined as the intermittent administration of full treatment courses of an antimalarial medicine to children aged three to 59 months in areas of highly seasonal transmission during the rainy season. SMC consists of the monthly administration of a full course of SPAQ treatment. The duration of treatment usually lasts four months. The objective is to prevent malarial illness by maintaining therapeutic antimalarial drug concentrations in the blood throughout the period of greatest malarial risk. The WHO recommends SMC with a combination of two antimalarial medicines, sulfadoxine-pyrimethamine + amodiaquine (SPAQ), in areas with highly seasonal malaria transmission in the Sahel sub-region of Africa, where *P. falciparum* is sensitive to both of these medicines. SMC was first recommended by the WHO in 2012, followed by the development of regional and national plans for its implementation [4]. During the high transmission period, SMC has been shown to reduce inpatient malaria deaths by an estimated 42% to 57% depending on the countries, and to reduce the number of confirmed outpatient malaria cases by an estimated 25% to 55% [5]. In addition, a recent retrospective study from Issiaka et al. demonstrated that the implementation of SMC was associated with a substantial reduction in hospital admissions and all-cause mortality in the health district of Ouelessebougou, Mali [6]. In Senegal, SMC has been used in children up to 10 years old based on the findings that the malaria burden was also high in the 5 to 10 years old group [7]. According to the WHO 2019 World Malaria Report, 31 million children in 12 countries in Africa’s Sahel sub-region were protected through SMC programs in 2018 Africa [1]. However, about 12 million children who could have benefited from this intervention were not covered.

Intermittent preventive treatment of infants (IPTi) is a course of antimalarial medicine delivered to infants using routine immunization services. Importantly, IPTi should not be deployed concomitantly with SMC [4]. IPTi treatment is given three times during the first year of life at approximately 8 to 10 weeks, 12 to 14 weeks, and nine months of age, corresponding to the routine vaccination schedule of the Expanded Programme on Immunization. The WHO recommends IPTi with sulfadoxine-pyrimethamine (SP) in areas with moderate to high malaria transmission in sub-Saharan Africa that have less than 50% prevalence of Pfdhps 540 mutation in the *P. falciparum* parasite, since Pfdhps 540 mutation is associated with SP resistance [8]. Administration is associated with a favorable safety and tolerability profile, and is simple, cost-effective, and accepted by health workers and communities, as indicated during the initial pilots in the early evaluation periods [9]. It has been confirmed that IPTi with SP has no negative effect on the protective efficacy of the Expanded Programme on Immunization vaccines [10,11]. A recent Cochrane review of IPTi by Esu et al. found that, although the effect varied over time and between drugs, the overall impact of IPTi was a 27% reduction of clinical malaria [12]. They concluded that IPTi with SP probably made little or no difference in all-cause mortality, but resulted in fewer episodes of clinical malaria, anemia, and parasitemia, and fewer hospital admissions. To date, Sierra Leone is the only country that has fully implemented the IPTi intervention.

Other preventive interventions have been trialed or piloted, including intermittent preventive treatment in school children (IPTsc), mass drug administration (MDA), and post-discharge malaria chemoprevention [13,14]. Post-discharge malaria chemoprevention being one of the most recently investigated interventions, we sought to better understand how it could be implemented. Post-discharge malaria chemoprevention is the intermittent administration of full treatment courses of antimalarials to children recovering from severe anemia. In Malawi, a randomized clinical trial of post-discharge malaria chemoprevention evaluated the effect of artemether lumefantrine (AL) administered one and two months after discharge to children under five years of age who had been admitted with severe anemia [15]. The intervention decreased the composite endpoint of death, severe anemia, or severe malaria by 31%. Similarly, a study in Kenya and Uganda demonstrated that the administration of dihydroartemisinin-piperaquine (DHA-PPQ) to children admitted for all-cause severe anemia at two, six, and 10 weeks after discharge reduced all-cause readmission or death by 35% [16].

Because the adoption and implementation of SMC has been fragmented and IPTi limited to Sierra Leone, we designed a survey to better understand how SMC and IPTi interventions are perceived in a selection of countries. In addition, as IPTi is delivered via the Expanded Programme on Immunization, we investigated the hurdles facing this program that could impact the potential implementation of IPTi. Lastly, as clinical trials are currently investigating the impact of providing intermittent administration of full courses of treatment of antimalarials to children recovering from severe anemia, we sought to better understand the management of children under five years of age hospitalized for severe anemia.

## 2. Materials and Methods

### 2.1. Study Setting

To understand in-country approaches to malaria chemoprevention in children, eight countries in Africa were selected based on their past and current experiences with various malaria chemoprevention strategies, as well as some countries that did not implement any intervention. Table 1 summarizes which interventions have been implemented or trialed in each country included in the study.

**Figure 1:**
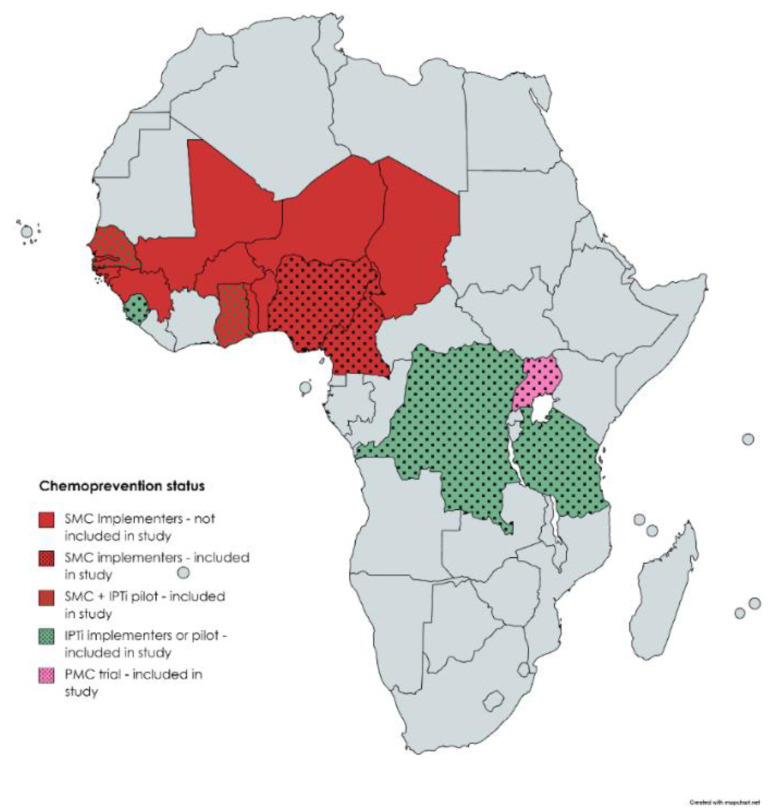
Chemoprevention geographical representation, including interventions that are currently implemented or piloted.

These countries were also selected because of their geographic diversity within Africa and their varying population size. Figure 1 provides a geographical view of the countries currently implementing or having piloted the various chemoprevention interventions. The dotted font is indicative of the countries that were included in this study. Participants from all countries were asked questions about their vaccination programs and how they manage severe anemia patients.

### 2.2. Key Informant Interviews

The first arm of this survey consisted of interviews with people selected for their first-hand knowledge about chemoprevention, as well as their role in decision-making processes at the national level. These people are referred to as key informants. Research participants were selected based on their involvement in malaria management, and included National Malaria Control Programme (NMCP) coordinators or staff members, key opinion leaders (KOLs), such as clinicians or professors, and in-country malaria organization representatives. Sampling was done through a mix of purposive and snowball method. Participation in the survey was voluntary, and no incentive or compensation was offered to participants. 

Five discussion guides were produced by Medicines for Malaria Venture and are provided in a supplementary file. The list of questions in each guide was adapted to the country’s past and current experience with malaria chemoprevention strategies. All interviews were conducted in French or English by phone by one of the authors (C.A.), with notes taken during the interview (no audio recording). 

### 2.3. Health Center Managers, Community Health Workers, and Parents Interviews

Seven discussion guides were generated by Medicines for Malaria Venture and shared with its field partner, Sanisphere (https://www.sanisphere.com, accessed on 10 May 2021). The discussion guides are available as supplementary files. Sanisphere is a market research agency specialized in healthcare working exclusively in low- and middle-income countries. Each discussion guide was tailored to the target respondent (health center manager, community health workers, parents) and the type of intervention (IPTi, SMC, extended SMC, vaccination campaign, management of severe anemia). Separate local ethics clearance was obtained for each country and each site by the field partner. The objective of the study was described before each interview, and written informed consent was sought from each participant. Confidentiality was assured at all stages of the study, and permission was asked for tape recording. The study did not involve patients, and data on patient characteristics were provided only in the aggregate. As such, there was no institutional review board involved in approving the research per the European Pharmaceutical Market Research Association (EphMRA) code of conduct and the British Health Business Intelligence Association (BHBIA) guidelines [17,18].

At the field level, the recruitment and interview of health center managers, community health workers, and parents was undertaken by Sanisphere. Respondents were recruited through convenience sampling in districts and health centers where children receive SMC and/or participate in IPTi. Potential participants were approached either by phone or email. Screening questions were used to either qualify or disqualify respondents from participating in the interview. These questions were adapted to each country’s policy regarding SMC and IPTi implementation status, as specified in Table 1.

### 2.4. Data Analysis

For the key informant interviews, debriefing notes were generated by the moderator for each call/meeting. The 14 debriefing notes were structurally coded and entered in Excel to facilitate the analysis. Analysis was performed by the moderator, who conducted the interviews to ensure consistency in the analysis.

Content analysis of the transcripts from the interviews with health center managers, community health workers, and parents was performed by one researcher who systematically reviewed the 165 transcripts and entered and coded the data into Excel to facilitate the analysis. Coding themes were not identified in advance, but were derived from the data. The code book was integrated into the Excel file used for the analysis. A second researcher independently coded a random sample of 27 transcripts (10 health center managers, 10 community health workers, and seven parents). To judge coding reliability, Cohen’s kappa was used to examine the consistency between both coders. Findings of this analysis indicated that the coefficients for each variable examined were 0.72 for health center managers, 0.61 for community health workers, and 0.73 for parents, with an aggregated score of 0.69. Given that attaining a kappa value of 0.6 or higher is considered a substantial level of mutual agreement, we determined that our coding was reliable [19]. Cohen’s kappa score was not calculated for the key informant because of the low number of interviews. Participants did not provide feedback on the findings.

## 3. Results

For key informants, personalized introduction emails were sent by the authors to 32 potential key informant participants, outlining the scope of the project. Seven (21%) contacts declined to participate in the survey because they were not involved in malaria chemoprevention, but provided references and introductions to other key informants. Eleven (32%) contacts did not respond or were not able to participate in the timeframe allocated for that research. No one from Tanzania was interviewed, as it was not possible to schedule a time for the interviews during the period allocated to the study. A total of 14 contacts accepted the invitation and were interviewed, including six NMCP coordinators, two NMCP staff members, four professors from academic or research centers, and two local UNICEF representatives. The interviews took place from 19 June to 25 July 2019. One respondent in the Democratic Republic of Congo (DRC) provided feedback by email. Interviews lasted from 15 to 60 minutes, with an average of 33 min.

The final sample for the field survey consisted of 94 health center managers, 46 community health workers, and 25 parents, who were all interviewed in the health center they worked for or were associated with. The total number of respondents per type and per country is shown in Table 2. A detailed sample composition description of each target can be found in the Tables S1–S3 in Supplementary Materials. All interviews were conducted face-to-face in French or English. Interviews took place from 9 August 2019 to 28 September and lasted 45 min on average for health center managers and community health workers and 25 minutes on average for parents. The interviews were audio recorded and transcribed into English. Transcripts from the first four interviews in Nigeria and the DRC were shared with Medicines for Malaria Venture to serve as a pilot and provide further guidance to interviewers. The quality of the pilot interviews was satisfactory, and they were included in the final analysis. Transcripts were then received on a bi-weekly basis. No repeat interviews were carried out, and transcripts were not returned to participants for comments or corrections. A total of 165 transcripts were received and coded as described in the methodology section.

The number of participants per country is shown in Table 2.

### 3.1. Seasonal Malaria Chemoprevention

#### 3.1.1. Key Informants

The understanding of current SMC practices was investigated by interviewing a total of nine NMCP representatives, KOLs, and in-country partners from Cameroon, Ghana, Senegal, and Nigeria. According to the nine key informants, the SMC intervention implementation was facilitated by the fact that good results were obtained during trials and local pilots. Engagement of policy makers and community leaders was mentioned as the key driver of implementation and acceptance of the intervention. The hurdles encountered were mainly linked to adherence to the program and administration of the second and third doses of SPAQ, as well as limited funding in Nigeria. In Senegal, the extension of SMC to children five to 10 years of age was triggered by the early SMC trials, which demonstrated that children in this older age group were just as affected by malaria as the younger children. According to the key informants from other SMC-implementing countries interviewed for this survey, a similar extension to five to 10 years old did not occur mainly because of limited funding and the absence of a pilot study in their countries to demonstrate the same benefit in the older age category.

#### 3.1.2. Field Participants

Results from the interviews with 37 health center managers, 46 community health workers, and 20 parents in Cameroon, Ghana, Nigeria, and Senegal are presented below.

- Positive perception of SMC

**Figure 2:**
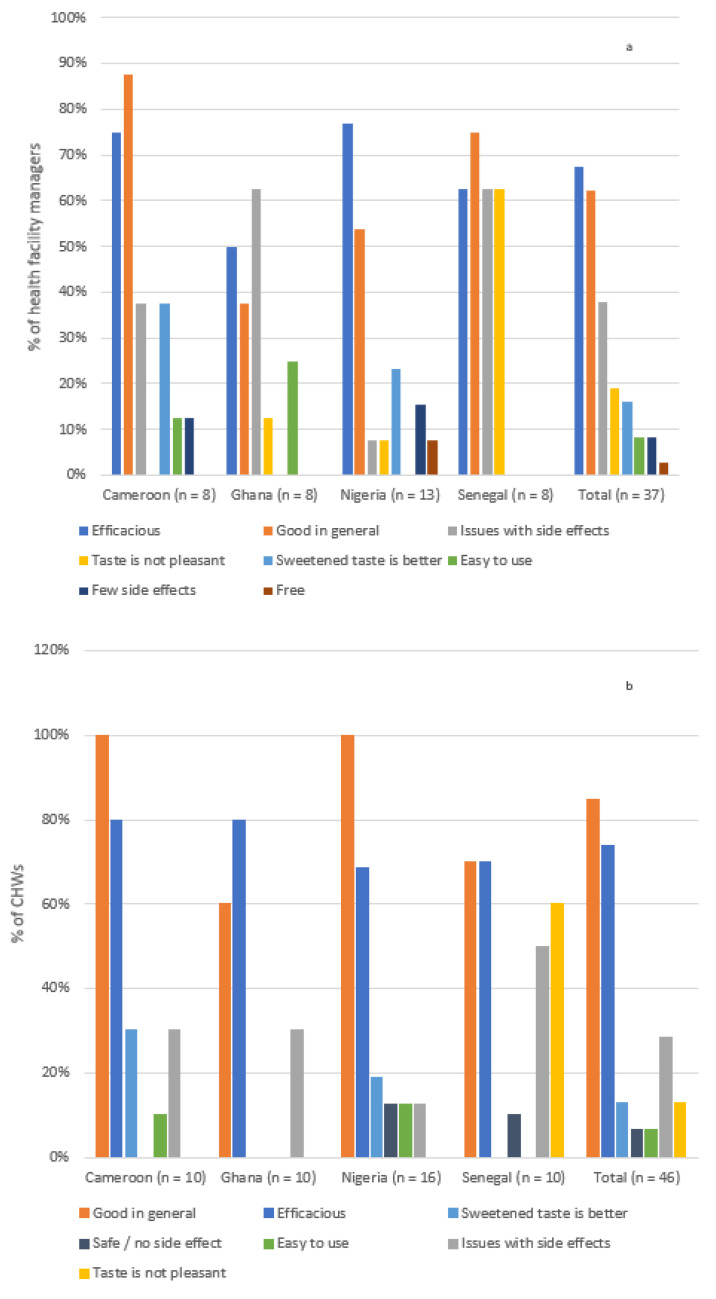
SPAQ perception by (**a**) health center managers and (**b**) community health workers.

SMC was generally positively perceived by the survey participants thanks to its efficacy in preventing malaria (Figure 2).

Samples of illustrative quotes are provided below.

Efficiency: “The drug is very effective. The reason I said this is because before the start of this chemoprevention, during raining season, facilities are usually congested with sick people. All of the beds for admission would have been occupied. But due to the effectiveness of the chemoprevention such congestion is now a thing of the past.” Health center manager, Nigeria

Cost: “SMC reduces expenses for us parents. There are too many mosquitoes here; without prevention it would be too expensive to treat children. Malaria access requires spending in health centers, yet prevention is free.” Mother of two children, Cameroon.

- Barriers to SMC implementation

**Figure 3:**
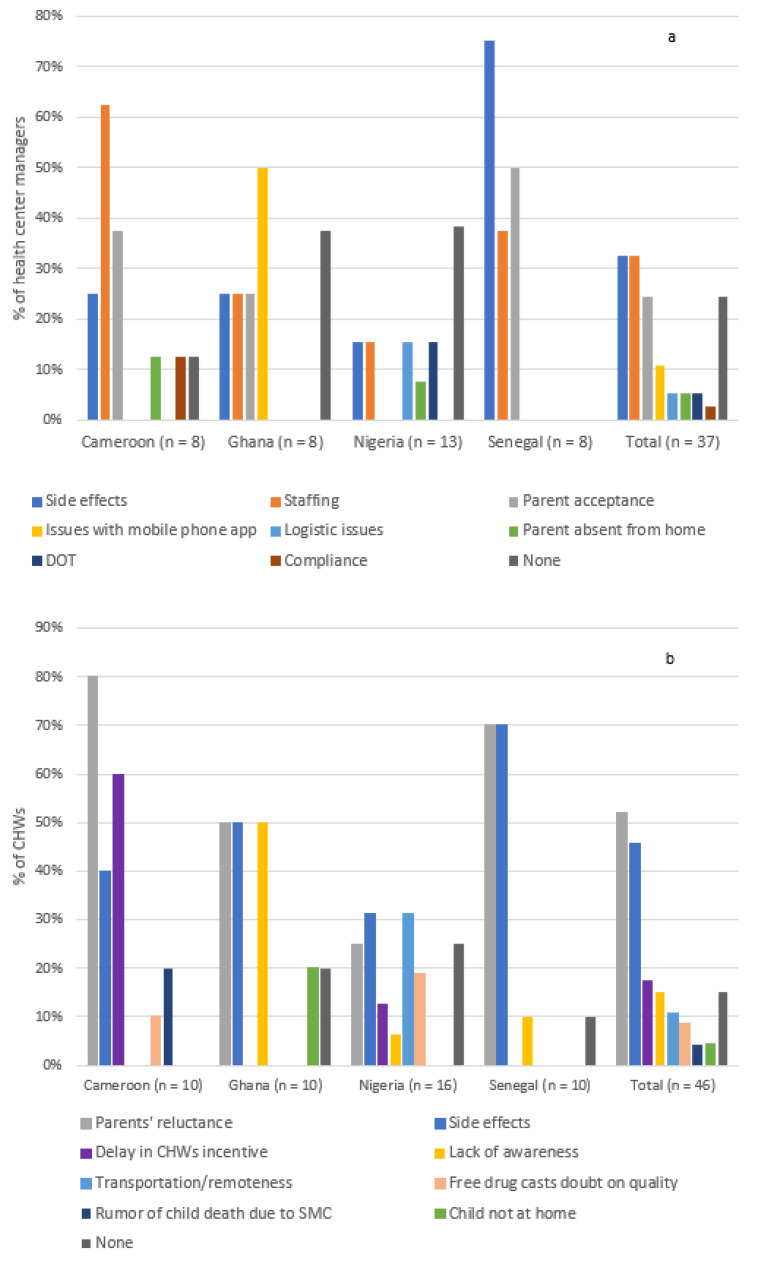
Barriers to SMC implementation at (**a**) the health center level and (**b**) the community health worker level.

The main barriers to SMC implementation mentioned by healthcare professionals were the side effects, driven by respondents from Senegal, a number of staffing issues, including lack of staff, transportation to households, and absence or delay in payment of incentives to staff, and poor acceptance from parents (Figure 3).

Adverse effect: “During the first day we had less problems, but on the second day, third day we had all the problems in the world because it was at that moment that the side effects started and we had many cases of refusal because the parents could not bear these effects in the children and they said that the drugs are not good. In the second passage, it was even worse, we had many cases of refusal; when we went to discuss with the parents, they said downright ‘no, all the children were sick’, we tried to talk to them but there was nothing to do.” Health center manager, Senegal.

- Areas for SMC improvement

**Figure 4:**
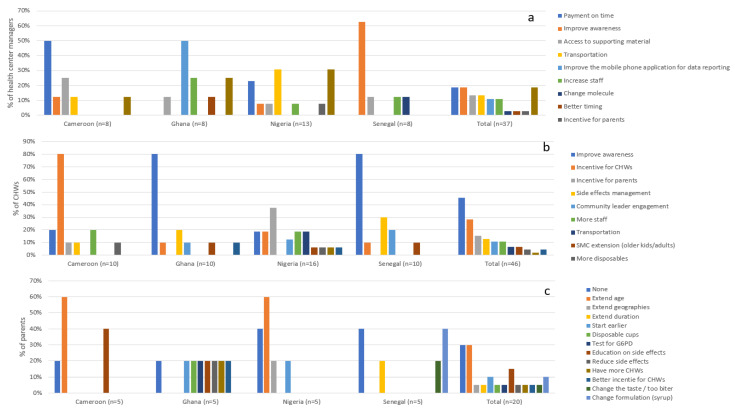
SMC improvements that could be implemented at (**a**) the health center manager level, (**b**) the community health worker level, and (**c**) the parent level.

The three main ways to improve SMC delivery according to healthcare professionals who participated in the survey are: (1) better education of parents to increase their acceptance of SMC (Senegal); (2) on time payment of incentives (Cameroon), offering a better incentive plan to cover the transportation fee, or offering a small incentive to parents (Nigeria and Ghana); and (3) improvement of the side effects associated with the treatment (Senegal). For parents involved in the survey, the main suggested improvements were extension of SMC (to older children and even adults and to regions that are not currently covered) and extended duration (coverage for a longer period than the current four months) (Figure 4). 

Several potential barriers to SMC were specifically investigated during the survey. These included issues around drug stocks, staffing and workload, inadequate coverage, and potential conflicts with other public health campaigns.

Availability of the drug: health center managers and community health workers who participated in this study anecdotally reported SPAQ out of stock situations. Most of these situations were corrected within 24 hours and had no impact on children’s malaria protection.

Staffing and workload: Health center managers with the lowest number of community health workers were those most in need of an increase in staff, requesting on average 36% more community health workers. Half of the community health workers who had participated in the survey said the workload associated with SMC was fine, while the other half thought it was too much. In terms of turnover, most parents who participated in the survey in Nigeria and Cameroon mentioned that community health workers varied frequently, while parents from Senegal and Ghana reported that it was steadier.

Coverage: According to health center managers and community health workers who participated in the survey, 88% and 92% of eligible children received the first dose of SMC each month, respectively. In terms of the second and third doses, an estimated 11% of children did not receive it, according to one health center manager. This number increased to 22% of children, according to community health workers. Respondents from Cameroon, Ghana, and Nigeria indicated that the main reasons for not providing SMC to children was absence or illness, while in Senegal it was parental refusal. A number of measures have been put in place by respondents to ensure that children in Cameroon, Ghana, and Nigeria receive the second and third doses of SMC, including providing advice to mothers, collecting empty blisters as proof of administration, and the use of recording cards to keep track of administration.

Coverage: “… without lying to you, officially almost all the children received. But unofficially, those who have actually received are few, given the demotivation of community health workers on the field. Hence, it can be estimated that 25% of children have not received their SPAQ dose.” Health center manager, Cameroon.

Conflicting campaigns: Lastly, although conflicting interventions were mentioned as a potential barrier, it was noted that SMC campaigns tend to run in isolation, according to most health center managers and community health workers interviewed. On the rare occasions when other interventions took place at the same time as SMC, these caused no conflict. Some anecdotal comments pointed to possible benefits of running net distribution, nutrition education programs, or vaccination campaigns at the same time as SMC.

### 3.2. Intermittent Preventive Treatment of Infants (IPTi)

IPTi was investigated by interviewing two key informant respondents from Sierra Leone, the only country where IPTi is currently routinely performed. In addition, experience with IPTi was investigated in the three countries that piloted IPTi, namely the DRC, Ghana, and Senegal, by interviewing two key informants per country. Finally, the general perception of IPTi was obtained from three key informant respondents from Cameroon and Uganda. In Sierra Leone, the main drivers for implementing IPTi were the very high mortality rate in infants and the absence of seasonal transmission that made SMC unsuitable. IPTi adoption was facilitated by the good perception of SP, thanks to the malaria prevention in pregnancy program and the important role played by community health workers in sensitizing the community to the intervention. The main hurdles were related largely to logistic issues, such as availability of clean water, drug shortages, nurse turnover, and poor attendance to Expanded Programme on Immunization visits.

The DRC, Ghana, and Senegal piloted IPTi, but decided not to implement it for the following reasons: lack of involvement of policy makers from the start of the process, logistics issues around synchronization with other programs, the belief of some healthcare professionals that infants are protected by their mothers’ antibodies at the beginning of life, unconvincing results at the end of the trial, fear of SP side effects, overexposure to SP in infants due to their mother receiving SP during pregnancy, absence of solutions past the age of one, and the gap between two vaccination programs, leading to a gap in coverage. Cameroon and Uganda did not pilot IPTi and do not plan to implement it, because they are concerned about resistance to SP. They foresee difficulties in integrating it into the Expanded Programme on Immunization­, the acceptance of parents is expected to be low, and the gap between two administrations does not provide full coverage. Nigeria is the only country that envisages implementing IPTi to reduce malaria burden in infants.

In addition to key informant interviews, IPTi was investigated by interviewing 15 health center managers and five parents in Sierra Leone. According to the former, the advantages of IPTi were its effectiveness in reducing the number of malaria cases, the absence of side effects, and the good supply of SP. The main barriers were the difficulties in preparing the drug, with crushing the tablets being the hardest and most time-consuming step of the treatment; the limited access to commodities, such as clean water and cups; and the lack of training of staff/nurses due to high turnover rates. Participating parents were largely satisfied with IPTi, because they perceived it as efficacious in protecting infants from malaria and having no side effects. The main areas for improvements highlighted were an increase in drug stocks to avoid having to return to the facility, access to clean water, and availability of a pediatric formulation.

### 3.3. The Expanded Programme on Immunization

**Figure 5:**
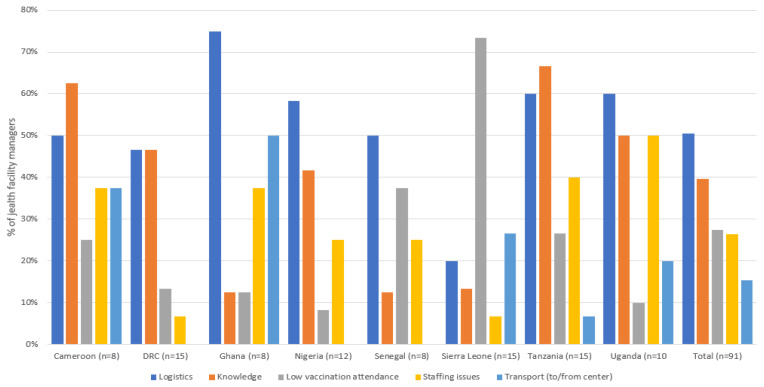
Main issues reported about the Expanded Programme on Immunization.

Perception, drivers, and barriers of the Expanded Programme on Immunization were investigated by interviewing 91 health center managers involved in the Expanded Programme on Immunization in all eight countries: Cameroon, the DRC, Ghana, Nigeria, Senegal, Sierra Leone, Tanzania, and Uganda. The Expanded Programme on Immunization was largely well-perceived by communities and parents, according to the health center managers who participated in the survey. The stated main hurdles fell into five broad categories: logistics-related issues, such as running out of stock or inadequate storage of product; lack of knowledge or understanding of the program by parents and lack of endorsement by community leaders; children not showing up; staffing issues, such as a lack of staff and high staff turnover; and transportation difficulties both for parents to come to the Expanded Programme on Immunization center and for staff to travel to communities (Figure 5). 

Reported attendance for vaccination visits ranged from 66% to 93% of eligible children at 10 weeks, 68% to 90% of eligible children at 14 weeks, and 54% to 88% at nine months. The measures put in place to increase attendance to Expanded Programme on Immunization visits by survey participants included having a system that tracks the no-shows, raising awareness amongst parents, developing outreach services, and providing an incentive to parents.

### 3.4. Post-Discharge Malaria Chemoprevention (PMC)

Post-discharge malaria chemoprevention is currently being investigated in several clinical trials in Uganda, Kenya, and Malawi. We evaluated the degree of knowledge and interest in this intervention amongst the 14 NMCPs, KOLs, and in-country malaria organizations included in our survey. Very little knowledge about this intervention was observed among all 14 participants, even in Uganda, where it has been evaluated in a clinical trial.

In addition, our evaluation shows that the 94 health center managers who participated in the research were largely unfamiliar with PMC. Therefore, the focus was on capturing some basic information about the management of patients under five years of age presenting with severe anemia who required hospitalization. Forty-three percent of the health center managers we interviewed said they either do not see or do not manage these patients. The remaining 57% who do take care of these patients claimed that they performed malaria tests in 94% of children, with 66% coming back positive. Transfusion was needed for 77% of children, with 86% of them receiving it as reported by our sample. The biggest issues faced by interviewed health center managers regarding the treatment of children under five years of age with severe anemia were parents’ financial difficulties, lack of blood for transfusion, artesunate out of stock, delay in seeking treatment, and poor adherence with the treatment.

## 4. Discussion

The efficacy and safety of malaria chemoprevention interventions in children and infants have largely been documented, and numerous publications of clinical trials results are available. There is, however, little literature on end users’ experiences outside of clinical trials. This qualitative study provides novel and valuable insights on the perceptions and attitudes towards SMC and IPTi from those who implement it in the field—health center managers, community health workers, and parents—as well as national representatives who are involved in setting up malaria elimination policy in their country.

Regarding SMC, the results from this study indicate that it is largely well-perceived by end users. The good efficacy and generally favorable safety profile, as well as the growing experience with this intervention, drove the positive perception. Similar findings have been reported by other researchers, including a recent publication of a qualitative study among parents and community health workers in the upper west region of Ghana, which demonstrated that the high acceptability of the SMC intervention was driven by the perception that SMC had helped reduce the prevalence of malaria [20]. The success of SMC at the end user level could not have been achieved without the early endorsement of the intervention by national stakeholders. Feedback obtained from key informant participants identified that robust clinical trial data at the country level as well as the involvement of local policy makers as early as possible in the exploration of the intervention facilitated its adoption. These are important findings for countries where SMC is not yet implemented. National programs willing to implement SMC in a country should make sure that key national stakeholders and policy makers are involved at the beginning of the project, and should leverage the good results obtained in other countries if local trials have not been conducted. Although an investigation of funding was not part of the scope of this survey, it must be noted that it is a critical element that impacts the uptake and rollout of any intervention. Finally, several countries are in the process of redefining their malaria epidemiological stratification. This exercise is important to define which areas are eligible for SMC implementation. Efficacy and cost effectiveness of SMC is highly linked to the seasonality of malaria transmission in the targeted areas.

Despite SMC’s high acceptability at the central and field levels, coverage remained below 100%, meaning that not all eligible children received chemoprotection, and not all who received it completed the full seasonal course. In our survey, health center managers and community health workers reported that 90% of eligible children received the first dose each month at best. The consequences of poor adherence to the SMC program can be dramatic, as it raises the risk of resistance to the drugs used and impairs the efficacy of the intervention [21]. Although numerous studies have tried to estimate adherence to SMC, it must be emphasized that, to date, there is little objective, quantitative data that confirm adherence (such as plasma drug levels) from areas where SMC is implemented as a routine program [20,21,22,23]. In the absence of such a standardized systematic measure of adherence, we investigated the causes for reduced adherence in order to identify measures that could increase access to SMC. Participants in our survey indicated that the main reasons for low coverage were child absenteeism, ailing children, or parental reluctance to allow their children to receive a drug. Community health workers participating in our survey mentioned the latter as the biggest hurdle. Work from other groups identified further causes for low coverage [22,23], including insufficient training of community distributors, inadequate supply of commodities, insufficient financial resources for remuneration, advocacy, and supervision, and parents forgetting to administer the second and third doses. There were anecdotal mentions of the drug being given to another child or saved for later, as well as parental reluctance to allow their children to receive the drug [20,23]. These findings illustrate the breadth of reasons that can prevent all eligible children from receiving SMC, and must be taken into consideration by policy makers and program heads who want to start or optimize SMC in their country.

The potential hurdles to SMC implementation are not limited to uptake or adherence issues. In their 2017 publication, Coldiron et al. identified logistical burdens, the use of preventive medication requiring a three-day course of therapy, and geographical difficulties as potential challenges to full SMC implementation [21]. Our survey also investigated stocking issues, staffing and workload issues, and conflicts with other campaigns as potential barriers to SMC. Stocking and potential conflicts with other campaigns were not reported as having an impact on SMC implementation, neither positive nor negative. Most of the out of stock situations lasted one day and were quickly resolved, and there was little overlap of SMC campaigns with other interventions. Staffing issues, on the contrary, were mentioned as an important barrier to SMC implementation, mainly not enough community health workers to perform the campaign, resulting in a heavier workload for those on the ground. Delay in payment of community health worker incentives was also mentioned as a demotivating factor, and increases staff turnover. These hurdles are important to take into consideration when implementation or extension of SMC is planned.

Regarding IPTi, results from participants based in Sierra Leone indicate that the intervention is largely accepted and integrated in the Expanded Programme on Immunization. Drivers of adoption of IPTi at the national level were the high mortality rate in infants, good efficacy data shown in early clinical trials, and the absence of a significant rainy season that prevented Sierra Leone from implementing SMC. These arguments were not sufficient for decision makers from the other countries included in our survey to decide to implement IPTi. The reasons for their reluctance ranged from fear of the emergence of resistance to SP, expected difficulties in synchronizing with the Expanded Programme on Immunization, unconvincing efficacy results, and gaps in coverage. These concerns are in line with the potential challenges identified and discussed in 2005 by O’Meara et al. [24]. Several potential adaptations can be envisaged to make IPTi more attractive to decision makers, including: extending IPTi beyond the third vaccination up until children reach two years of age; using a drug other than SP in areas where SP resistance is a concern; and synchronizing IPTi with interventions other than the Expanded Programme on Immunization to increase the number of drug doses. These adaptations will need to be trialed and endorsed by regulatory authorities before they can be rolled out. At the policy level, it is important to engage with local decision makers as early as possible in the process and to allow them to tailor the approach to their country’s needs. At the field level, parents and community health workers from Sierra Leone recognized the efficacy of the intervention in protecting their infants from malaria. The main difficulties at this level were related to logistics issues, such as access to water and crushing the tablets, and staffing issues related to high staff turnover and training needs.

Lastly, our survey provided some high-level information about the potential hurdles to the implementation of post-discharge malaria chemoprevention. Participants in our survey indicated that financial difficulties experienced by parents and poor adherence to treatments were two of the biggest issues they faced when treating patients under five years of age presenting with severe anemia. These two potential barriers will have to be addressed should PMC be implemented at a large scale, in addition to raising awareness about this intervention.

The main limitation of the interviews with key informants was the small number of participants. Caution should be used when generalizing the results of this subset of respondents to the whole malaria prevention field. To mitigate the impact of the small sample size, we targeted participants who were the most influential and knowledgeable. The survey with field-level participants also presents a number of limitations. Regarding the sample size and composition, the total number of respondents per country was limited, and not all regions or districts were represented. Although we interviewed respondents from a mix of regions and settings to mitigate geographical bias, caution should be used when generalizing the results of this subset of respondents to the entire malaria-endemic region of sub-Saharan Africa. In terms of methodology, as with any survey, our findings reflect the answers and views of participants that may be influenced by their recall and response bias. To mitigate this risk, all key informants were interviewed by one person to achieve greater uniformity in interpretation. As for the interviews with field-level participants, clear guidance was provided to interviewers to ensure that they obtained the most accurate information. None of the interviewers had a pre-existing relationship with the survey participants. Several interviewers were used to administer the questionnaire in the different countries, which could result in some interviewer-based country variation. Importantly, all respondents answered the same questionnaire, and interviewers were trained to avoid this type of bias. All interviewers were professional market researchers having at least 10 years of experience in healthcare market research. They all hold a diploma in healthcare, public health, or pharmacy at the Master’s or PhD level. Despite these limitations, we believe that the data presented here are valuable, as they provide some qualitative input on the experience with and perception of malaria chemoprevention interventions, and they represent real-life data typically not obtainable on a large scale.

## 5. Conclusions

This end user perspective study has provided new insights about the drivers and barriers for the adoption of various malaria chemoprevention interventions from the point of view of a select group of target respondents, key stakeholders, and end users. Wherever implemented, the chemoprevention interventions explored in our survey were perceived as valuable and efficacious in preventing malaria by survey participants. Logistical issues, such as staff shortages and out of stock situations, were identified as the biggest barrier to the full use of these interventions, highlighting the need for health system strengthening measures. Further expansions of these interventions, either to eligible geographies or broader age groups, could be envisaged, providing that health systems are able to absorb the increased workload and the provision of the sufficient drug is secured, that country-generated data on the positive impact of these interventions are available, and that the expansions are endorsed by normative bodies.

## Figures and Tables

**Table 1:** Chemoprevention intervention per country.

Country	SMC ^1^	Ext SMC ^2^	IPTi	PMC
Cameroon	Yes	No	No	No
Democratic Republic of Congo (DRC)	No	No	Pilot	No
Ghana	Yes	No	Pilot	No
Nigeria	Yes	No	No	No
Senegal	Yes	Yes	Pilot	No
Sierra Leone	No	No	Yes	No
Tanzania	No	No	Pilot	No
Uganda	No	No	No	Trial

^1^ Years of SMC implementation are: 2016 in Cameroon, 2015 in Ghana, 2014 in Nigeria, and 2013 in Senegal; ^2^ Ext SMC: extended seasonal chemoprevention to the five to 10 year age group.

**Table 2:** Chemoprevention intervention per country.

Country	Health Center Managers	Community Health Workers	Parents	Key Informants	Total
Cameroon	8	10	5	2	25
DRC	16	0	0	2	18
Ghana	8	10	5	2	25
Nigeria	13	16	5	3	37
Senegal	8	10	5	2	25
Sierra Leone	15	0	5	2	22
Tanzania	15	0	0	0	15
Uganda	11	0	0	1	16
Total	94	46	25	14	179

## Data Availability

The dataset supporting the conclusions of this article is included within the article (and its Supplementary Materials).

## Supplementary Materials

The following are available online at https://www.mdpi.com/article/10.3390/tropicalmed6020075/s1, Table S1: Sample composition Health Center Managers, Table S2: Sample composition Community Health Workers, Table S3: Sample composition Parents.

## Abbreviations

AL: artemether lumefantrine; BHBIA: British Health Business Intelligence Association; DHA-PPQ: dihydroartemisinin-piperaquine; DRC: the Democratic Republic of Congo; EphMRA: European Pharmaceutical Market Research Association; IPTi: intermittent preventive treatment of infants; IPTp: intermittent preventive treatment of pregnant women; IPTsc: intermittent preventive treatment in school children; KOLs: key opinion leaders; MDA: mass drug administration; NMCP: National Malaria Control Program; SMC: seasonal malaria chemoprevention; SP: sulfadoxine-pyrimethamine; SPAQ: sulfadoxine-pyrimethamine + amodiaquine; WHO: the World Health Organization.
