# Supplementary material for: Perception of Malaria Chemoprevention Interventions in Infants and Children in Eight Sub-Saharan African Countries: An End User Perspective Study
Source: Trop Med Infect Dis. 2021 May 11;6(2):75. doi: 10.3390/tropicalmed6020075 (PMC8163176; doi:10.3390/tropicalmed6020075)
Supplement: Supplementary file 1 [file tropicalmed-06-00075-s001.zip › tropicalmed-1197343-supplementary.pdf]

Supplementary information: Questionnaire central level participants

Module 1. Introduction – all groups

Thank you for according me the time to speak with you today. My name is Céline Audibert and I am the Market Research Director at Medicines for Malaria Ventures (MMV). The purpose of our discussion is to better understand how malaria chemoprevention in infants and children is currently done in your country; what led to the selected approaches, and any development you expect in the future. Before we start with specific questions about this topic, I would like to mention that:

- The research will involve interviewing you on a series of questions that may take up to 60 minutes.
- Any answer that you give us will be combined with answers from many other people, so that your responses will not make you identifiable in our reporting.
- *We would like to audio record the interview – We will use this audio recording to help us write up a transcript of the interview (a written account of what was said) and we will then delete the audio recording.*
- *We will use these transcripts to help us write our report, however we will remove any information that identifies you in the transcript.*
- Your participation in the interview is voluntary, and you can decide to stop at any point without explanation. There will be no penalty for doing so.
- Your answer to this survey will help to provide evidence on current status of and needs for malaria chemoprevention in infant and children in your country.
- There are no right or wrong answers to any of these questions.
- Is there anything else you would like to know about the survey?

Do you agree to participate in the study today?

Module 2. Respondent background – all groups

[We will most likely have the information about the participant already, but the aim of these questions is to get familiar with speed of speech and pronunciation before diving into the specific questions]

Could you please provide some background information about yourself, like the number of years you have been involved in the fight against malaria, your education, the number of years in your current position etc.

Module 3. Current approach to chemoprevention in infants and children – all groups

[The aim of this section is to validate the information we obtained through desk research. It gives the opportunity to identify any gap between the publicly available sources of information such as WHO report, Malaria indicator Survey reports, National Malaria Strategic plans and PMI's malaria operational plan with what is actually happening in the country]

- How are infants and children protected against malaria in your country?
    - Avoid the discussion on ITN, the focus is drug based prevention.
    - If IPTi is not mentioned, ask the participant about his/her knowledge about this prevention method.

    - For SMC, validates the treatment protocol they apply: age range, molecule used, number of cycle, how are the treatments delivered.
- Which guidelines, if any, do you follow, for both IPTi and SMC?
    - If different guidelines than the WHO, ask participant to explain why?
- How is the success of these interventions measured, if at all? Where are the data centralized? Who is in charge of analyzing them? At which frequency?
    - Probe on DHIS2, DHS, any other data collection technique, any support from external organizations in analyzing the data (MEASURE)

Module 4. About current implementation of IPTi – IPTi users only

We will focus on IPTi for a moment, and then discuss other malaria chemoprevention measures in children.

- What made you/your country decide to implement IPTi?
- What was the compelling information that helped you take this decision?
- How did you interpreted the WHO Policy guidelines for implementing IPTi? Are there parts that are confusing? Do you know the estimated level of SP resistance in your country/districts?
- What are the levels of sub-national uptake of IPTi? Is it directly related to the attendance of EPI visits, or are there other restrictions to uptake among districts?
- How much additional time does delivery of IPTi take, on top of a routine EPI visit?
- Is there more adherence to IPTi by EPI visit? Are children more likely to receive SP at specific vaccine visits?
- What are additional costs for IPTi delivery, other than procurement of SP? Training, supervision, additional staff?
- How successful has this IPTi program been?
    - What proportion of infants are currently covered with IPTi?
    - What was the target coverage? What are the reasons for over/under achieving the target?
    - What needs to happen to increase the number of infants covered?
- What are the drivers and barriers to IPTi implementation?
    - At the central level (NMCP, MoH)
        - Probe on the issue of resistance to SP
    - At the procurement level (Global Fund, PMI, other…)
        - Probe on possible supply shortage of SP
    - At the district level
    - At the CHW level
- What would be your advice for a country that hasn't implemented IPTi yet (what are the do's and don'ts)?

Module 5. About future use of IPTi – IPTi users only

- How sustainable is the program? How long do you plan to have this intervention in place?
- Could IPTi delivery be adaptable to a 3-day regimen, taking home the additional 2-day treatment (i.e. for implementation of another 3-day antimalarial combination in areas of high SP-resistance)?

- What difference would current IPTi implementers perceive if they were to adopt dispersible SP (thanks to new sources of WHO-pre-qualified SP dispersible formulations that are used in SMC) compared to the original non-child-friendly SP formulations used in IPTi).

Module 6. About current implementation of SMC – SMC users only

We will now talk about seasonal malaria chemoprevention in children.

- What made you/your country decide to implement SMC?
- What was the compelling information that helped you take this decision?
- Confirm the current coverage of SMC (ie validate the information obtained through desk research) in terms of age range, geographical coverage and season
  - For those that extended the use to children >5yrs, what did they base their decision on?
  - For those using SMC in <5y only, what are their thoughts about expanding to older children?
- Confirm that SPAQ is the drug procured for SMC.
  - What is their level of concerns regarding SP/AQ resistance? Any actions they have put in place to monitor the resistance? Do they envisage switching to another drug?
- How successful has this intervention been?
  - What proportion of children are currently covered with SMC?
  - What was the target coverage? What are the reasons for over/under achieving the target?
  - What needs to happen to increase the number of infants covered?
- What are the drivers and barriers to SMC implementation?
  - At the central level (NMCP, MoH)
    - Probe on the issue of resistance to SP/AQ
  - At the procurement level (Global Fund, PMI, other…)
    - Probe on possible supply shortage of SPAQ
  - At the district level
  - At the CHW level

Module 7. About future use of SMC – SMC users only

- How sustainable is the intervention? How long do you plan to have this intervention in place?
- Do you have any plan to adapt the existing SMC program?
  - If yes, probe on age range, drug choice, extended seasonal coverage.
  - If not, ask why.

Module 8. Not IPTi users

[Separate set of questions for those who trialed IPTi and those who have no experience with IPTi at all]

Never used IPTi:

- What have they heard about IPTi? Are they envisaging implementation in any parts of their country? Reasons why/why not?
- If they plan to implement it, what is the timeframe for implementation? What drivers could speed up or delay implementation? What has been the limiting factor?
- What are their thought about extending the age range for IPTi?
- If they don't intend to implement, what is the rational? What would need to happen for them to implement IPTi?

Trialed IPTi

- What has been their experience with IPTi? In how many districts/centers did they pilot it?
- What has been the outcome of the pilot? What worked well and what didn't work well?
- Why did they decide not to implement IPTi in their countries? What would need to happen for them to implement it?
- What are the levels of sub-national uptake of IPTi? Is it directly related to the attendance of EPI visits, or are there other restrictions to uptake among districts?
- How much additional time does delivery of IPTi take, on top of a routine EPI visit?
- Is there more adherence to IPTi by EPI visit? Are children more likely to receive SP at specific vaccine visits?
- What are additional costs for IPTi delivery, other than procurement of SP? Training, supervision, additional staff?

Module 9. Not SMC users

- Are they envisaging SMC implementation in any parts of their country? Reasons why/why not?
- If they plan to implement it, what is the timeframe for implementation? What drivers could speed up or delay implementation? What has been the limiting factor?
- What are their thought about extending the age range for SMC?
- If they don't intend to implement, what is the rational? What would need to happen for them to implement SMC?

Module 10. Other interventions

[Questions below will be asked to countries that have experience with each intervention]

Post-discharge chemoprevention

- Your country is currently trialing post-discharge chemoprevention. What motivated you/your country to test this intervention? Can you provide more details on the intervention (where it is implemented, patient selection, drug used etc…)?
- What is the outcome of the trial so far *(remind respondent to not share confidential information)*?
- How does this intervention fit with the rest of the chemoprevention plan?
- Do they plan to roll out this intervention at the country level? If yes: how?, if not: why not?

IPTc

- Your country is currently trialing IPTc. What motivated you/your country to test this intervention? Can you provide more details on the intervention (where it is implemented, patient selection, drug used etc…)?
- What is the outcome of the trial so far *(remind respondent to not share confidential information)*?
- How does this intervention fit with the rest of the chemoprevention plan?
- Do they plan to roll out this intervention at the country level? If yes: how?, if not: why not?

Supplementary information: Questionnaire Healthcare Center Managers – DRC, Tanzania

Screener

Does or did this healthcare center run malaria chemoprevention interventions?

- **Yes:** Continue
- **No:** Continue

Do you treat infants under 1 year old at this health center?

- **Yes:** Continue
- **No:** Thank & end

What is your position/title at this health center?

Do you manage the healthcare facility?

- **Yes:** Continue with introduction
- **No:** Could you please refer me to the healthcare center manager or the most senior person who is currently onsite?

BASIC INFORMATION

**Respondent ID**	
**Healthcare center Name**	
**Healthcare center Address**	
**Healthcare center type (Public/Private)**	
**Respondent Position/Title**	
**Number of Patients (children) catered for by the healthcare center (on average a week)**	
**Interviewer**	
**Date of Interview**	
**Time Interview Started**	

Introduction of Health Center Manager (5 minutes)

Please tell me a little about yourself.

- How long have you worked at this health center?
- How many years of experience do you have including work at other health facilities?
- What is your education?
- Can you please tell me about your day to day/ routine activities in the hospital/ clinic?

Catchment:

1. In the community that your health center covers approximately how many children are below 1 year?

2. Roughly what % of the health center's infant/child patients visit the center regularly and what % visit for the first-time?

3. What do you think is the furthest/longest a child has to travel to reach your health center?

4. How far away is the next health center from here?

5. What is typically the children's parents' economic status? (*Check for* ***no income/low/ mid/high income:*** *for example:* ***no income:*** *unemployed/subsistence farmer,* ***low-mid income:*** *farmer, trader/market seller, employed in factory,* ***mid-high income:*** *employed in office/company owner)*

Expanded Program on Immunization (EPI):

6. Are all children (under 1 year) in your community covered by the EPI program (Expanded Program on Immunization)? If no: why not?

7. Can you describe in your own words how EPI is performed at your health center?

8. What would you say are the biggest problems that your healthcare center faces with EPI? **(unprompted to capture their spontaneous/top of mind concerns) PROBE THOUROUGHLY** 'what else', 'what else' etc.

9. Is there anything that you would like to see improved regarding EPI which would improve the results of infant immunization in your community? **(unprompted to capture their spontaneous/top of mind concerns) PROBE THOUROUGHLY** 'what else', 'what else' etc.

10. How does your health center run the EPI program? Is it on a rolling basis, or do you have dedicated times during the week/month?

11. Out of all the under 1 year olds living in the health center's catchment area what % do you think attend EPI?

12. What are the infants ages when they attend EPI? ***(In question 13, 14, 15 please use the appropriate infant ages that the respondent told you in question 12)***

13. What % of **XXX** old infants attend EPI?

14. What % of **XXX** weeks old infants attend EPI?

15. What % of **XXX** months old infants attend EPI?

16. What do you think are the main reasons for missed visits?

17. Does your health center have measures in place to help with attendance of infants? **If yes:** What are these measures?

18. How much time does your staff spend on average per child per EPI visit?

19. How are vaccinations in general seen in the community?

20. How is EPI perceived by parents?

Intermittent preventive treatment for infants (IPTi):

21. Have you heard about an intervention called IPTi (Intermittent preventive treatment for infants)?
    If yes:
    - Please describe what you know about IPTi?

22. Were you involved in the IPTi trial that was conducted in your country?
    If yes:
    - When did the IPTi trial take place?
    - What type of training did you receive?
    - Approximately, how many infants were covered during the trial?
    - What % of infants received IPTi at their first vaccination visit (infants of 10 weeks)?
    - What % of infants received IPTi at their second vaccination visit (infants of 14 weeks)?
    - What % of infants received IPTi at their third vaccination visit (infants of 9 months)?
    - How much time did IPTi add to a regular vaccination visit?
    - What were the good things about the IPTi trial, if any? **(unprompted to capture their spontaneous/top of mind concerns)**
    - Did the trial increase attendance to EPI?
    - Did it reduce the number of malaria cases?
    - What were the difficulties with the IPTi trial, if any? **(unprompted to capture their spontaneous/top of mind concerns)**
    - How time consuming was it?
    - Did you have enough clean water at the health center to administer IPTi?
    - Was is difficult or time consuming to split and crush the tablet?
    - Did you observe any side effect of IPTi?
    - In your opinion why was the IPTi intervention not continued?

    If no:
    - Do you think IPTi would be useful for your community?
    - What benefits do you see in IPTi?
    - What disadvantages do you see in IPTi?

Post-discharge malaria chemoprevention (PDC)

23. Out of the total infants/children (0-10 years) your center sees, what % suffer from malaria? Is this percentage similar throughout the year or are there seasonal differences?

24. On average in one month, how many children below 5 are admitted to your health center for severe anemia?

25. What is approximately the % of these children (below 5) with anemia that receive a malaria diagnosis test?

26. Of the children (below 5) tested, how many are positive for malaria?

27. For those positive for malaria, how are they treated & managed?
    - How many require transfusion?
    - How many receive transfusion?
    - On average how long do the patients stay in center?
    - How frequently do you see these patients come back to the health center within one month?
    - When these patients are discharged, are they given preventive antimalaria treatment?
      If yes: Which preventive treatment are they given and for how long?
    - Thinking about managing those patients with malaria what would you say are the biggest problems in managing them?

28. Have you heard about post-discharge malaria chemoprevention trials done in Malawi, Uganda and Kenya?
    If yes: What have you heard about these trials? What is your opinion about these trials?

29. In your view, do you think there are “false beliefs” that are common in the community regarding malaria chemoprevention?
    If yes: Please tell me what these false beliefs are.

Thank you very much for your answers

Supplementary information: Questionnaire Healthcare Center Managers – Sierra Leone

Screener

Does this healthcare center run IPTi (Intermittent preventive treatment for infants)?

- **Yes:** Continue
- **No:** Thank & end

What is your position/title at this health center?

Do you manage the healthcare facility?

- **Yes:** Continue with introduction
- **No:** Could you please refer me to the healthcare center manager or the most senior person who is currently onsite?

BASIC INFORMATION

**Respondent ID**	
**Healthcare center Name**	
**Healthcare center Address**	
**Healthcare center type (Public/Private)**	
**Respondent Position/Title**	
**Number of Patients (children) catered for by the healthcare center (on average a week)**	
**Interviewer**	
**Date of Interview**	
**Time Interview Started**	

Introduction of Health Center Manager (5 minutes)

Please tell me a little about yourself.

- How long have you worked at this health center?
- How many years of experience do you have including work at other health facilities?
- What is your education?
- Can you please tell me about your day to day/ routine activities in the hospital/ clinic?

Catchment/Coverage:

1. In the community that your health center covers approximately how many children are below 1 year?

2. Roughly what % of the health center's infant/child patients visit the center regularly and what % visit for the first-time?

3. What do you think is the furthest/longest a child has to travel to reach your health center?

4. How far away is the next health center from here?

5. What is typically the children's parents' economic status? (*Check for* ***no income/low/ mid/high income:*** *for example:* ***no income:*** *unemployed/subsistence farmer,* ***low-mid income:*** *farmer, trader/market seller, employed in factory,* ***mid-high income:*** *employed in office/company owner)*

<u>Expanded Program on Immunization (EPI):</u>

6. Are all children below 1 year covered by the EPI program (Expanded Program on Immunization)? **If no:** why not?

7. Can you describe in your own words how EPI is performed at your health center?

8. How does your health center run the EPI program? Is it on a rolling basis, or do you have dedicated times during the week/month?

9. Out of all the under 1 year olds living in the health center's catchment area what % do you think attend EPI?

10. What % of 10 weeks old infants attend EPI?

11. What % of 14 weeks old infants attend EPI?

12. What % of 9 months old infants attend EPI?

13. What do you think are the main reasons for missed visits?

14. Does your health center have measures in place to help with attendance of infants? **If yes:** What are these measures?

<u>Intermittent preventive treatment for infants (IPTi):</u>

15. When did your health center start IPTi?

16. Since you started IPTi approximately how many infants received IPTi?

17. Do all children that are part of the EPI program receive SP (Sulfadoxine/pyrimethamine)? **If no:** why not?

18. Can you describe in your own words how your healthcare center runs IPTi (Intermittent preventive treatment for infants)?

19. What would you say are the biggest problems that your healthcare center faces with IPTi? **unprompted to capture their spontaneous/top of mind concerns) PROBE THOUROUGHLY** 'what else', 'what else' etc.

20. Is there anything that you would like to see improved regarding IPTi? **unprompted to capture their spontaneous/top of mind concerns) PROBE THOUROUGHLY** 'what else', 'what else' etc.

21. Please tell me how you were trained on IPTi?

22. When was your training and how long did the training last?

23. Who attended the training?

24. What was the content of the training?

25. Was there anything that was missing in your training but if it had been included you would have been better prepared for running the IPTi program? **If yes:** Please describe what you would have liked to be included in your training and why.

26. Would you say that a refresher training course would be useful to you? **If yes:** why?

27. Is there more adherence to IPTi by EPI visit? Are children more likely to receive SP at specific vaccine visits (e.g. more likely on the first visit or more likely on the last visit etc.)?

28. How much additional time does delivery of IPTi take, on top of a routine EPI visit?

29. What is the most time-consuming step?

30. What is the water source at their center (SP needs to be diluted in water)?

31. How often is your center out of clean water?

32. Approximately how often does an infant not receive SP because of lack of clean water?

33. How frequently do parents come with their own clean water?

34. Can you please tell me what your opinion is with regards to the medication used for IPTi: SP (sulfadoxine-pyrimethamine)? **FREE FLOW DISCUSSION. TO AID DISCUSSION, USE THE FOLLOWING PROBES**
    - Quality
    - Availability/Supply
    - Ease of Use
    - Effectiveness/Resistance
    - Side effects if any
    - Price/Cost
    - Any other aspect?

35. How difficult is it to split and crush the tablet?

36. How useful would it be to have a pediatric formulation for SP, and why?

37. How do you manage the SP stocks for IPTi?

38. How frequently do you experience out of stock situations for IPTi?

39. What are the main causes for the out of stock situations?

40. How long is your health center typically out of stock?

41. What would need to happen to reduce the out of stock situation?

42. What side effects linked to SP do you observe, if any?

43. What do parents think of IPTi?

44. Do they express concerns that their infant received a drug while he/she is not sick?
    If yes: What % of parents would you say express concern that the infant is receiving medication even though he/she is not sick?

Post-discharge malaria chemoprevention (PDC)

45. Out of the total infants/children (0-10 years) your center sees, what % suffer from malaria? Is this percentage similar throughout the year or are there seasonal differences?

46. On average in one month, how many children below 5 are admitted to your health center for severe anemia?

47. What is approximately the % of these children (below 5) with anemia that receive a malaria diagnosis test?

48. Of the children (below 5) tested, how many are positive for malaria?

49. For those positive for malaria, how are they treated & managed?
    - How many require transfusion?
    - How many receive transfusion?
    - On average how long do the patients stay in center?
    - How frequently do you see these patients come back to the health center within one month?
    - When these patients are discharged, are they given preventive antimalaria treatment?
      If yes: Which preventive treatment are they given and for how long?

- Thinking about managing those patients with malaria what would you say are the biggest problems in managing them?

50. Have you heard about post-discharge malaria chemoprevention trials done in Malawi, Uganda and Kenya?
    If yes: What have you heard about these trials? What is your opinion about these trials?

51. In your view, do you think there are “false beliefs” that are common in the community regarding malaria chemoprevention?
    If yes: Please tell me what these false beliefs are.

Thank you very much for your answers

Supplementary information: Questionnaire Parents – Sierra Leone

Screener

How old are your children?

- **Under 1:** Continue
- **1-2:** Continue
- **Over 2:** Thank & end

Did your children receive all of the required vaccinations (at 10 weeks, 14 weeks and 9 months)?

- **Yes:** Continue
- **No:** Did they receive at least one of the required vaccinations?
  - **Yes:** Continue
  - **No:** Thank & end

Have any of your children received malaria chemoprevention at the same time as the vaccination (IPTi)? Was this given as a tablet that was crushed and dissolved in water? **(caution – parents are sometimes confused about what type of pill infants receive, and think it is paracetamol)**

- **Yes:** Continue
- **No:** Thank & end

BASIC INFORMATION

**Respondent ID**	
**Healthcare center Name**	
**Healthcare center Address where parent was recruited**	
**Healthcare center type (Public/Private)**	
**Relationship to child (mother, father)**	
**Number of children**	
**Interviewer**	
**Date of Interview**	
**Time Interview Started**	

Introduction of Parents (10 minutes)

Please tell me a little about yourself.

- What is your profession and education?
- How many children do you have?
- How old are each of your children?
- Have all of your children received malaria chemoprevention? Malaria chemoprevention is typically given at the same time as vaccination, and in the form of a tablet that is crushed before being dissolved in water. **If not all receive:** Why not all?
- How often would you say you get malaria?
- What about your children? How often do they get malaria?

__**Malaria Chemoprevention**__

1. Can you describe in your own words the process/journey of how your child(ren) receive malaria chemoprevention?

2. How did you hear about malaria chemoprevention?

3. What was explained to you about malaria chemoprevention?

4. Did your child miss one or more of the 3 vaccinations?

   If yes:
   - What was the reason why your child missed any of the vaccinations? **Probe in detail:** (distance to the health facility, infant sick, other…)?

5. What do you think of malaria chemoprevention in general?

6. What do you like about malaria chemoprevention? **Probe 'what else & why', 'what else & why'**

7. Why do you say this?

8. What don't you like about malaria chemoprevention? **Probe 'what else & why', 'what else & why'**

9. Why do you say this?

10. What are your suggestions to make malaria chemoprevention better, what do you think can be improved?

11. Why do you say this?

12. Did your child have issues swallowing the medicine?
    If yes: Why do you think it had problems swallowing (e.g. taste, pieces that were not fully diluted)
13. How do you feel about the preparation of the medication, is there anything that bothers you about how it is prepared? **Probe (health professional crushing tablet, diluting in water etc.)**

14. For how long do you feel your child is protected from malaria thanks to malaria chemoprevention?

15. Has your child ever experienced any side effects when they took malaria chemoprevention?

If yes: Which side effects did they experience? Will you give your child malaria chemoprevention again or recommend to other parents to give to their children even though your child suffered side effects?
   If yes: Why do you say this?

16. Have you seen any difference with the number of malaria episodes that your child experiences when they take malaria chemoprevention compared to before they started taking malaria chemoprevention? **If yes:** what difference did you observe?

17. When your child suffers from a malaria episode, what do you do?

18. Do you have them tested to confirm malaria or do you give them malaria medication without the test?

19. In your opinion what should be done differently for malaria chemoprevention?

20. In your view, do you think there are “false beliefs” that are common in your community regarding malaria chemoprevention?
    If yes: Please tell me what these false beliefs are.

Thank you very much for your answers

Supplementary information: Questionnaire Healthcare Center Managers – Uganda

Screener
Do you treat infants under 1 year old at this health center?

- **Yes:** Continue
- **No:** Thank & end

What is your position/title at this health center?
Do you manage the healthcare facility?

- **Yes:** Continue with introduction
- **No:** Could you please refer me to the healthcare center manager or the most senior person who is currently onsite?

BASIC INFORMATION

**Respondent ID**	
**Healthcare center Name**	
**Healthcare center Address**	
**Healthcare center type (Public/Private)**	
**Respondent Position/Title**	
**Number of Patients (children) catered for by the healthcare center (on average a week)**	
**Interviewer**	
**Date of Interview**	
**Time Interview Started**	

Introduction of Health Center Manager (5 minutes)
Please tell me a little about yourself.

- How long have you worked at this health center?
- How many years of experience do you have including work at other health facilities?
- What is your education?
- Can you please tell me about your day to day/ routine activities in the hospital/ clinic?

Catchment:

1. In the community that your health center covers approximately how many children are below 1 year?

2. Roughly what % of the health center's infant/child patients visit the center regularly and what % visit for the first-time?

3. What do you think is the furthest/longest a child has to travel to reach your health center?

4. How far away is the next health center from here?

5. What is typically the children's parents' economic status? (*Check for **no income/low/ mid/high income:** for example: **no income:** unemployed/subsistence farmer, **low-mid income:** farmer, trader/market seller, employed in factory, **mid-high income:** employed in office/company owner)*

<u>Expanded Program on Immunization (EPI):</u>

6. Are all children (under 1 year) in your community covered by the EPI program (Expanded Program on Immunization)? If no: why not?

7. Can you describe in your own words how EPI is performed at your health center?

8. What would you say are the biggest problems that your healthcare center faces with EPI? **(unprompted to capture their spontaneous/top of mind concerns) PROBE THOUROUGHLY** 'what else', 'what else' etc.

9. Is there anything that you would like to see improved regarding EPI which would improve the results of infant immunization in your community? **(unprompted to capture their spontaneous/top of mind concerns) PROBE THOUROUGHLY** 'what else', 'what else' etc.

10. How does your health center run the EPI program? Is it on a rolling basis, or do you have dedicated times during the week/month?

11. Out of all the under 1 year olds living in the health center's catchment area what % do you think attend EPI?

12. What are the infants ages when they attend EPI? ***(In question 13, 14, 15 please use the appropriate infant ages that the respondent told you in question 12)***

13. What % of **XXX** old infants attend EPI?

14. What % of **XXX** weeks old infants attend EPI?

15. What % of **XXX** months old infants attend EPI?

16. What do you think are the main reasons for missed visits?

17. Does your health center have measures in place to help with attendance of infants? **If yes:** What are these measures?

18. How much time does your staff spend on average per child per EPI visit?

19. How are vaccinations in general seen in the community?

20. How is EPI perceived by parents?

Post-discharge malaria chemoprevention (PDC)

21. Out of the total infants/children (0-10 years) your center sees, what % suffer from malaria? Is this percentage similar throughout the year or are there seasonal differences?

22. On average in one month, how many children below 5 are admitted to your health center for severe anemia?

23. What is approximately the % of these children (below 5) with anemia that receive a malaria diagnosis test?

24. Of the children (below 5) tested, how many are positive for malaria?

25. For those positive for malaria, how are they treated & managed?
    - How many require transfusion?
    - How many receive transfusion?
    - On average how long do the patients stay in center?
    - How frequently do you see these patients come back to the health center within one month?
    - When these patients are discharged, are they given preventive antimalaria treatment?
      If yes: Which preventive treatment are they given and for how long?
    - Thinking about managing those patients with malaria what would you say are the biggest problems in managing them?

26. Have you heard about post-discharge malaria chemoprevention trials done in Malawi, Uganda and Kenya?
    If yes: What have you heard about these trials? What is your opinion about these trials?

Intermittent preventive treatment for infants (IPTi):

27. Have you heard about an intervention called IPTi (Intermittent preventive treatment for infants)?
    If yes:
    - Please describe what you know about IPTi?
    - Do you think it would be useful for your community?
    - What benefits do you see in IPTi?
    - What disadvantages do you see in IPTi?

28. In your view, do you think there are “false beliefs” that are common in the community regarding malaria chemoprevention?
    If yes: Please tell me what these false beliefs are.

Thank you very much for your answers

Supplementary information: Questionnaire Community Health Workers – Nigeria, Cameroon, Senegal, Ghana

Screener

Are you currently working on/involved with an SMC (Seasonal Malaria Chemoprevention) campaign?

- **Yes:** Continue
- **No:** Thank & end

Do you or have you administered SMC to infants and children?

- **Yes:** Continue
- **No:** Thank & end

What is your position/title?

BASIC INFORMATION

**Respondent ID**	
**Healthcare center Name**	
**Healthcare center Address or address where CHW was recruited**	
**Healthcare center type (Public/Private)**	
**Respondent Position/Title**	
**Number of Patients (children) under responsibility of this CHW**	
**Interviewer**	
**Date of Interview**	
**Time Interview Started**	

Introduction of CHWs (5 minutes)

Please tell me a little about yourself.

- How long have you worked as a CHW?
- What was your motivation to become a CHW and contribute to the fight against malaria?
- What is your education?
- Can you please tell me about your day to day/ routine activities as a CHW?

Coverage:

1. In total how many children under 5 years are you responsible for?

2. How about older children, how many children aged 5-10 are you responsible for? *(If respondent has trouble answering then ask how many children above 5 years of age)*

3. How do you feel about the number of households that you are responsible for (too many households, too few households, just about right)?

4. Could you please tell me why you say that?

5. From your experience what percentage of households with children under the age of 5 also have children aged 5-10 years living in the same household?

6. How long does it take you to distribute SPAQ to all the children under your responsibility? **Cameroun, Ghana, Nigeria only:** If you had to include the children aged 5 to 10 years old, how much longer would you need, if at all?

7. Approximately what % of your time working in your capacity as a CHW do you spend travelling to/from the children that are under your responsibility?

8. What is the furthest/longest you need to travel to visit a child under your responsibility?

9. How do you feel about the time/effort you have to spend travelling to reach the children?

10. What mode of transportation do you use to go from house to house (walk, bicycle, motorbike, bus, car etc.)?

11. Please tell me your thoughts about reaching the children by door-to-door visits (advantages & disadvantages of the door-to-door method)?

12. Could you think of a better way than door-to-door visits of how the children could get their doses for SMC?

13. From the households you visit, what is typically the children's parents' economic status? (*Check for* ***no income/low/ mid/high income:*** *for example:* ***no income:*** *unemployed/subsistence farmer,* ***low-mid income:*** *farmer, trader/market seller, employed in factory,* ***mid-high income:*** *employed in office/company owner)*

<u>**Seasonal Malaria Chemoprevention (SMC)**</u>

14. Can you describe in your own words how you run an SMC (Seasonal Malaria Chemoprevention) campaign?

15. What would you say are the biggest problems that you face with SMC? **unprompted to capture their spontaneous/top of mind concerns) PROBE THOUROUGHLY** 'what else', 'what else' etc.

16. Is there anything that you would like to see improved regarding SMC which would improve the results of SMC campaigns? What changes would you like to see that would improve the way you administer/distribute SMC? **unprompted to capture their spontaneous/top of mind concerns) PROBE THOUROUGHLY** 'what else', 'what else' etc.

17. Thinking of the last SMC campaign, when did it start?

18. Were there any other campaigns running at the same time and conflicting with the SMC campaign? (for example, EPI: Expanded Program on Immunization)
    If yes:
    - Which conflict did you experience?
    - What were the consequences?
    - What was done to limit or counteract the consequences of the conflict?

    If no:
    - Did you experience a conflict in the past during other SMC campaigns?
        If yes:
        - What kind of conflict did you experience?
        - What were the consequences?
        - What was done to limit or counteract the consequences of the conflict?

19. Apart from other healthcare related campaigns were there any other events that influenced or conflicted with the last SMC campaign (for example: religious celebration, field work, unrest etc…).
    If yes: Please describe the conflicts and the consequences for the SMC campaign.

20. Please tell me how you were trained on SMC?

21. How long did the training last?

22. Was there anything that was missing in your training but if it had been included you would have been better prepared for your role as an CHW? **If yes:** Please describe what you would have liked to be included in your training and why.

23. Would you say that a refresher training course would be useful to you? **If yes:** why?

24. Can you please tell me what your opinion is with regards to the medication used for SMC: SPAQ (sulfadoxine-pyrimethamine + amodiaquine)? **FREE FLOW DISCUSSION. TO AID DISCUSSION, USE THE FOLLOWING PROBES**
    - Quality
    - Price/Cost
    - Effectiveness
    - Any other aspect?

25. Thinking about Availability/Supply of SPAQ, how do you manage the SPAQ stock?

26. Where do you receive supply from?

27. How do you receive the SPAQ (pick-up, delivery etc.)?

    If pick-up: How far do you have travel to get SPAQ supply (in minutes/hours or kilometers)?

If delivery: Who delivers it to you?

28. How often do you get/receive supply (daily, several times a week, weekly, once a month etc.)?

29. What quantities of SPAQ do you stock per time (stock for how many treatments)?

30. How do you store the SPAQ?

31. Did you experience out of stock during this SMC campaign?
    If yes:
    - Why did you run out of stock?
    - For how long were you out of stock?
    - How many children do you estimate missed their dose because of out of stock?

32. Do you expect an out of stock situation during the rest of the SMC campaign?

33. Did you experience out of stock during the past SMC campaign?
    If yes:
    - Why did you run out of stock?
    - For how long were you out of stock?
    - How many children do you estimate missed their dose because of out of stock?

34. In an average month of the last SMC campaign, out of all the children under your responsibility what % of children received SPAQ?

    If not all received: Please tell me the reasons why not all children received SPAQ.

35. What % of all the children under your responsibility do you estimate did not receive SPAQ during the last SMC campaign?

36. In your opinion what were the reasons why these children did not receive SMC (for example: children not at home because they have to help their parents in field, or are away visiting family, parents do not want to give medication to a healthy child etc.).

37. How frequently do you estimate children miss the 2nd and 3rd doses of SMC?

38. Do you have any measures in place that make sure that children receive the 2nd and 3rd doses?
    If yes: What are these measures?

39. How often would you say is there a gap of more than 4 weeks between 2 SMC distributions?

40. What are the reasons for this gap?

41. In your view, do you think there are “false beliefs” that are common in the community regarding malaria chemoprevention?
    If yes: Please tell me what these false beliefs are.

Thank you very much for your answers

Supplementary information: Questionnaire Parents – Nigeria, Cameroon, Senegal, Ghana

Screener

How old are your children?

- **Under 5:** Continue
- **5-10:** Continue
- **Over 10:** Thank & end

Have any of your children received malaria chemoprevention (Seasonal Malaria Chemoprevention)?

- **Yes:** Continue
- **No:** Thank & end

BASIC INFORMATION

**Respondent ID**	
**Healthcare center Name**	
**Healthcare center Address or address where parent was recruited**	
**Healthcare center type (Public/Private)**	
**Relationship to child (mother, father)**	
**Number of children**	
**Interviewer**	
**Date of Interview**	
**Time Interview Started**	

Introduction of Parents (10 minutes)

Please tell me a little about yourself.

- How long have you lived in this area?
- What is your profession and education?
- How many children do you have?
- How old are each of your children?
- Do all of your children receive malaria chemoprevention? **If not all receive:** Why not all?
- How often would you say you get malaria?
- What about your children? How often do they get malaria?

Seasonal Malaria Chemoprevention (SMC)

1. Can you describe in your own words the process/journey of how your child(ren) receives malaria chemoprevention (Seasonal Malaria Chemoprevention)?

2. How did you hear about malaria chemoprevention?

3. What was explained to you about malaria chemoprevention?

4. How many times does the CHW (Community Health Worker) visit your house to give you the medication for malaria chemoprevention (1 time in the last four months, 2 times in the last four months etc.)?

5. Do they come every month? **If yes:** For how many months in a row did they come every month?

6. Are there any other useful things apart from malaria chemoprevention that the CHW talks to you about? **Probe** on nutrition recommendations, or discussion around vaccination

7. Do you think that you ever missed the CHW visit?

   If yes:
   - How do you know that you missed it?
   - Did the CHW come back at another time to compensate for the missed visit or did they only come back one month later?
   - Did they leave the dose for your child with your neighbor who gave it to you?

8. What was the reason why you missed the CHW visit (e.g. children working in the field, vacation, visiting family, other…)?

9. Is it always the same CHW that visits you or are there different ones?

10. Who administers malaria chemoprevention to your child on the first day (confirm whether the CHWs administer the drug or leave it for the parents to do it)?

11. Who administers malaria chemoprevention to your child on the second and third days?

    If parent administers:
    - What instructions did the CHW give you in terms of administering the doses?
    - Is it easy to remember that you need to give the dose to your child?
    - Have you ever forgotten to administer the dose to your child?
      If yes: What did you do (e.g. did not give dose at all, gave dose later (ask how much later)?
    - How often would you say that you forget to give your child the dose?
    - How are the doses packaged that the CHW gives you?
    - Did you ever lose the doses, or did the doses ever spoil because they became wet etc.?
    - Have you ever given the 2nd or 3rd dose to someone other than your child?
      If yes: Who did you give the dose to? Why did you do that?

12. What do you think of malaria chemoprevention in general?

13. What do you like about malaria chemoprevention?

14. Why do you say this?

15. What don't you like about malaria chemoprevention?

16. Why do you say this?

17. What are your suggestions to make malaria chemoprevention better, what do you think can be improved?

18. Why do you say this?

19. Has your child ever experienced any side effects when they took malaria chemoprevention?

    If yes: Which side effects did they experience? Will you give your child malaria chemoprevention again or recommend to other parents to give to their children even though your child suffered side effects?

    If yes: Why do you say this?

20. Have you seen any difference with the number of malaria episodes that your child experiences when they take malaria chemoprevention compared to before they started taking malaria chemoprevention? **If yes:** what difference did you observe?

21. When your child suffers from a malaria episode, what do you do?

22. Do you have them tested to confirm malaria or do you give them malaria medication without the test?

23. What do you like about the CHW visits?

24. Why do you say this?

25. What don't you like about the CHW visits?

26. Why do you say this?

27. In your view, do you think there are "false beliefs" that are common in your community regarding malaria chemoprevention?
    If yes: Please tell me what these false beliefs are.

Thank you very much for your answers

Supplementary information: Questionnaire Healthcare Center Managers – Senegal, Ghana

Screener

Does this healthcare center run SMC (Seasonal Malaria Chemoprevention) campaigns?

- **Yes:** Continue
- **No:** Thank & end

Do you treat infants under 1 year old at this health center?

- **Yes:** Continue
- **No:** Thank & end

What is your position/title at this health center?

Do you manage the healthcare facility?

- **Yes:** Continue with introduction
- **No:** Could you please refer me to the healthcare center manager or the most senior person who is currently onsite?

BASIC INFORMATION

**Respondent ID**	
**Healthcare center Name**	
**Healthcare center Address**	
**Healthcare center type (Public/Private)**	
**Respondent Position/Title**	
**Number of Patients (children) catered for by the healthcare center (on average a week)**	
**Interviewer**	
**Date of Interview**	
**Time Interview Started**	

Introduction of Health Center Manager (5 minutes)

Please tell me a little about yourself.

- How long have you worked at this health center?
- How many years of experience do you have including work at other health facilities?
- What is your education?
- Can you please tell me about your day to day/ routine activities in the hospital/ clinic?

Catchment:

1. In the community that your health center covers approximately how many children are there that are below 1 year, how many 1-5 and how many 5-10 year old?

2. Can you please tell me on average how many children below the age of 5 does your health center see in a typical week?

3. How about older children, how many children aged 5-10 does your center cater for on average in one week? *(If respondent has trouble answering then ask how many children above 5 years of age)*

4. Roughly what % of the health center's infant/child patients visit the center regularly and what % visit for the first-time?

5. What do you think is the furthest/longest a child has to travel to reach your health center?

6. How far away is the next health center from here?

7. What is typically the children's parents' economic status? (*Check for **no income/low/ mid/high income:** for example: **no income:** unemployed/subsistence farmer, **low-mid income:** farmer, trader/market seller, employed in factory, **mid-high income:** employed in office/company owner)*

<u>**Expanded Program on Immunization (EPI):**</u>

8. Are all children (under 1 year) in your community covered by the EPI program (Expanded Program on Immunization)? **If no:** why not?

9. Can you describe in your own words how EPI is performed at your health center?

10. What would you say are the biggest problems that your healthcare center faces with EPI? **(unprompted to capture their spontaneous/top of mind concerns) PROBE THOUROUGHLY** 'what else', 'what else' etc.

11. Is there anything that you would like to see improved regarding EPI which would improve the results of infant immunization in your community? **(unprompted to capture their spontaneous/top of mind concerns) PROBE THOUROUGHLY** 'what else', 'what else' etc.

12. How does your health center run the EPI program? Is it on a rolling basis, or do you have dedicated times during the week/month?

13. Out of all the under 1 year old living in the health center's catchment area what % do you think attend EPI?

14. What are the infants ages when they attend EPI? ***(In question 15, 16, 17 please use the appropriate infant ages that the respondent told you in question 14)***

15. What % of ***XXX*** old infants attend EPI?

16. What % of ***XXX*** weeks old infants attend EPI?

17. What % of ***XXX*** months old infants attend EPI?

18. What do you think are the main reasons for missed visits?

19. Does your health center have measures in place to help with attendance of infants?
    If yes: What are these measures?

20. How much time does your staff spend on average per child per EPI visit?

21. How are vaccinations in general seen in the community?

22. How is EPI perceived by parents?

Intermittent preventive treatment for infants (IPTi):

23. Have you heard about an intervention called IPTi (Intermittent preventive treatment for infants)?
    If yes:
    - Please describe what you know about IPTi?

24. Were you involved in the IPTi trial that was conducted in your country?
    If yes:
    - When did the IPTi trial take place?
    - What type of training did you receive?
    - Approximately, how many infants were covered during the trial?
    - What % of infants received IPTi at their first vaccination visit (infants of 10 weeks)?
    - What % of infants received IPTi at their second vaccination visit (infants of 14 weeks)?
    - What % of infants received IPTi at their third vaccination visit (infants of 9 months)?
    - How much time did IPTi add to a regular vaccination visit?
    - What were the good things about the IPTi trial, if any? **(unprompted to capture their spontaneous/top of mind concerns)**
    - Did the trial increase attendance to EPI?
    - Did it reduce the number of malaria cases?
    - What were the difficulties with the IPTi trial, if any? **(unprompted to capture their spontaneous/top of mind concerns)**
    - How time consuming was it?
    - Did you have enough clean water at the health center to administer IPTi?
    - Was is difficult or time consuming to split and crush the tablet?
    - Did you observe any side effect of IPTi?
    - In your opinion why was the IPTi intervention not continued?

    If no:
    - Do you think IPTi would be useful for your community?
    - What benefits do you see in IPTi?
    - What disadvantages do you see in IPTi?

<u>Seasonal Malaria Chemoprevention (SMC)</u>

25. Can you describe in your own words how your healthcare center runs an SMC (Seasonal Malaria Chemoprevention) campaign?

26. What would you say are the biggest problems that your healthcare center faces with SMC? **unprompted to capture their spontaneous/top of mind concerns) PROBE THOUROUGHLY** 'what else', 'what else' etc.

27. What about the CHWs, what problems do you think they have with SMC? **(unprompted to capture their spontaneous/top of mind concerns) PROBE THOUROUGHLY** 'what else', 'what else' etc.

28. Is there anything that you would like to see improved regarding SMC which would improve the results of SMC campaigns? **unprompted to capture their spontaneous/top of mind concerns) PROBE THOUROUGHLY** 'what else', 'what else' etc.

29. What about the CHWs, in your opinion, what would help them to achieve better results? **(unprompted to capture their spontaneous/top of mind concerns) PROBE THOUROUGHLY** 'what else', 'what else' etc.

30. Thinking of the last SMC campaign that you ran at this center, when did it start?

31. Were there any other campaigns running at the same time at your center and conflicting with the SMC campaign? (for example, EPI: Expanded Program on Immunization)
    If yes: Which conflict did you experience?
    - What were the consequences?
    - What was done to limit or counteract the consequences of the conflict?

    If no: Did you experience a conflict in the past during other SMC campaigns?
    If yes: what kind of conflict did you experience?
    - What were the consequences?
    - What was done to limit or counteract the consequences of the conflict?

32. On a more positive note, were there any synergies between SMC and other healthcare related campaigns? **To help the discussion:** Did your health center, for example, link malnutrition assessment or vaccination check with the SMC campaign?
    If yes: What activities did you link with SMC?

33. Apart from other healthcare related campaigns were there any other events that influenced or conflicted with the last SMC campaign (for example: religious celebration, field work, unrest etc…).
    If yes: Please describe the conflicts and the consequences for the SMC campaign.

34. Can you please tell me what your opinion is with regards to the medication used for SMC: SPAQ (sulfadoxine-pyrimethamine + amodiaquine)? **FREE FLOW DISCUSSION. TO AID DISCUSSION, USE THE FOLLOWING PROBES**
    - Quality
    - Availability/Supply - How does your health center manage the SPAQ stock? Where do you receive supply from? How often do you receive supply etc.?
    - Price/Cost
    - Effectiveness
    - Any other aspect?

35. Thinking about the most recent SMC campaign that your health center conducted, how many CHW were involved?

36. Would you say that this is the ideal number of CHWs needed to run a successful SMC campaign?

    If no: In your opinion, what is the ideal number of CHWs involved in an SMC campaign? Why do you say this?

    If yes: Why do you say this?

37. Who is in charge of the CHWs for SMC?

38. How often do the CHWs receive training?

39. How does your health center manage the SPAQ stock?

40. How do the CHWs get the SPAQ supply?

41. How regularly do CHWs come to replenish their stocks?

42. Thinking of the last SMC campaign did your health center experience out of stock?

    If yes:
    - What was the reason for running out of stock?
    - For how long did your center not have any stock?
    - Approximately, how many children missed their dose?

    If no:
    - Did you experience out of stock during any of the past SMC campaigns?

      If yes:
        - What was the reason for running out of stock?
        - For how long did your center not have any stock?
        - Approximately, how many children missed their dose?

    If the SMC campaign is still running: Do you expect an out of stock situation during the current SMC campaign?

43. In an average month of the last SMC campaign, out of all the children that visited your health center what % of children received SPAQ?

    If not all received: Please tell me the reasons why not all children that visited your health center receive SPAQ.

44. What % of all the children living in the catchment area of your health center do you estimate did not receive SPAQ during the last SMC campaign?

45. In your opinion what were the reasons why these children did not receive SMC (for example: children not at home because they have to help their parents in field, are away visiting family, parents do not want to give medication to a healthy child etc.).

46. How frequently do you estimate children miss the 2nd and 3rd doses of SMC?

47. Do you have any measures in place that make sure that children receive the 2nd and 3rd doses?
    If yes: What are these measures?

Post-discharge malaria chemoprevention (PDC)

48. Out of the total infants/children (0-10 years) your center sees, what % suffer from malaria? Is this percentage similar throughout the year or are there seasonal differences?

49. On average in one month, how many children below 5 are admitted to your health center for severe anemia?

50. What is approximately the % of these children (below 5) with anemia that receive a malaria diagnosis test?

51. Of the children (below 5) tested, how many are positive for malaria?

52. For those positive for malaria, how are they treated & managed?
    - How many require transfusion?
    - How many receive transfusion?
    - On average how long do the patients stay in center?
    - How frequently do you see these patients come back to the health center within one month?
    - When these patients are discharged, are they given preventive antimalaria treatment?
      If yes: Which preventive treatment are they given and for how long?
    - Thinking about managing those patients with malaria what would you say are the biggest problems in managing them?

53. Have you heard about post-discharge malaria chemoprevention trials done in Malawi, Uganda and Kenya?
    If yes: What have you heard about these trials? What is your opinion about these trials?

54. In your view, do you think there are “false beliefs” that are common in the community regarding malaria chemoprevention?
    If yes: Please tell me what these false beliefs are.

Thank you very much for your answers

Supplementary information: Questionnaire Healthcare Center Managers – Nigeria, Cameroon

Screener

Does this healthcare center run SMC (Seasonal Malaria Chemoprevention) campaigns?

- **Yes:** Continue
- **No:** Thank & end

Do you treat infants under 1 year old at this health center?

- **Yes:** Continue
- **No:** Thank & end

What is your position/title at this health center?

Do you manage the healthcare facility?

- **Yes:** Continue with introduction
- **No:** Could you please refer me to the healthcare center manager or the most senior person who is currently onsite?

BASIC INFORMATION

**Respondent ID**	
**Healthcare center Name**	
**Healthcare center Address**	
**Healthcare center type (Public/Private)**	
**Respondent Position/Title**	
**Number of Patients (children) catered for by the healthcare center (on average a week)**	
**Interviewer**	
**Date of Interview**	
**Time Interview Started**	

Introduction of Health Center Manager (5 minutes)

Please tell me a little about yourself.

- How long have you worked at this health center?
- How many years of experience do you have including work at other health facilities?
- What is your education?
- Can you please tell me about your day to day/ routine activities in the hospital/ clinic?

Catchment:

1. In the community that your health center covers approximately how many children are there that are below 1 year, how many 1-5 and how many 5-10 year old?

2. Can you please tell me on average how many children below the age of 5 does your health center see in a typical week?

3. How about older children, how many children aged 5-10 does your center cater for on average in one week? *(If respondent has trouble answering then ask how many children above 5 years of age)*

4. Roughly what % of the health center's infant/child patients visit the center regularly and what % visit for the first-time?

5. What do you think is the furthest/longest a child has to travel to reach your health center?

6. How far away is the next health center from here?

7. What is typically the children's parents' economic status? (*Check for **no income/low/ mid/high income:** for example: **no income:** unemployed/subsistence farmer, **low-mid income:** farmer, trader/market seller, employed in factory, **mid-high income:** employed in office/company owner)*

<u>Expanded Program on Immunization (EPI):</u>

8. Are all children (under 1 year) in your community covered by the EPI program (Expanded Program on Immunization)?

9. Can you describe in your own words how EPI is performed at your health center?

10. What would you say are the biggest problems that your healthcare center faces with EPI? **(unprompted to capture their spontaneous/top of mind concerns) PROBE THOUROUGHLY** 'what else', 'what else' etc.

11. Is there anything that you would like to see improved regarding EPI which would improve the results of infant immunization in your community? **(unprompted to capture their spontaneous/top of mind concerns) PROBE THOUROUGHLY** 'what else', 'what else' etc.

12. How does your health center run the EPI program? Is it on a rolling basis, or do you have dedicated times during the week/month?

13. Out of all the under 1 year olds living in the health center's catchment area what % do you think attend EPI?

14. What are the infants ages when they attend EPI? ***(In question 15, 16, 17 please use the appropriate infant ages that the respondent told you in question 14)***

15. What % of ***XXX*** old infants attend EPI?

16. What % of ***XXX*** weeks old infants attend EPI?

17. What % of ***XXX*** months old infants attend EPI?

18. What do you think are the main reasons for missed visits?

19. Does your health center have measures in place to help with attendance of infants?
    If yes: What are these measures?

20. How much time does your staff spend on average per child per EPI visit?

21. How are vaccinations in general seen in the community?

22. How is EPI perceived by parents?

Post-discharge malaria chemoprevention (PDC)

23. Out of the total infants/children (0-10 years) your center sees, what % suffer from malaria? Is this percentage similar throughout the year or are there seasonal differences?

24. On average in one month, how many children below 5 are admitted to your health center for severe anemia?

25. What is approximately the % of these children (below 5) with anemia that receive a malaria diagnosis test?

26. Of the children (below 5) tested, how many are positive for malaria?

27. For those positive for malaria, how are they treated & managed?
    - How many require transfusion?
    - How many receive transfusion?
    - On average how long do the patients stay in center?
    - How frequently do you see these patients come back to the health center within one month?
    - When these patients are discharged, are they given preventive antimalaria treatment?
      If yes: Which preventive treatment are they given and for how long?
    - Thinking about managing those patients with malaria what would you say are the biggest problems in managing them?

28. Have you heard about post-discharge malaria chemoprevention trials done in Malawi, Uganda and Kenya?
    If yes: What have you heard about these trials? What is your opinion about these trials?

Seasonal Malaria Chemoprevention (SMC)

29. Can you describe in your own words how your healthcare center runs an SMC (Seasonal Malaria Chemoprevention) campaign?

30. What would you say are the biggest problems that your healthcare center faces with SMC? **unprompted to capture their spontaneous/top of mind concerns) PROBE THOUROUGHLY** ‘what else’, ‘what else’ etc.

31. What about the CHWs, what problems do you think they have with SMC? **(unprompted to capture their spontaneous/top of mind concerns) PROBE THOUROUGHLY** ‘what else’, ‘what else’ etc.

32. Is there anything that you would like to see improved regarding SMC which would improve the results of SMC campaigns? **unprompted to capture their spontaneous/top of mind concerns) PROBE THOUROUGHLY** ‘what else’, ‘what else’ etc.

33. What about the CHWs, in your opinion, what would help them to achieve better results? **(unprompted to capture their spontaneous/top of mind concerns) PROBE THOUROUGHLY** ‘what else’, ‘what else’ etc.

34. Thinking of the last SMC campaign that you ran at this center, when did it start?

35. Were there any other campaigns running at the same time at your center and conflicting with the SMC campaign? (for example, EPI: Expanded Program on Immunization)
    If yes: Which conflict did you experience?
    - What were the consequences?
    - What was done to limit or counteract the consequences of the conflict?

    If no: Did you experience a conflict in the past during other SMC campaigns?
    If yes: what kind of conflict did you experience?
    - What were the consequences?
    - What was done to limit or counteract the consequences of the conflict?

36. On a more positive note, were there any synergies between SMC and other healthcare related campaigns? **To help the discussion:** Did your health center, for example, link malnutrition assessment or vaccination check with the SMC campaign?
    If yes: What activities did you link with SMC?

37. Apart from other healthcare related campaigns were there any other events that influenced or conflicted with the last SMC campaign (for example: religious celebration, field work, unrest etc…).
    If yes: Please describe the conflicts and the consequences for the SMC campaign.

38. Can you please tell me what your opinion is with regards to the medication used for SMC: SPAQ (sulfadoxine-pyrimethamine + amodiaquine)? **FREE FLOW DISCUSSION. TO AID DISCUSSION, USE THE FOLLOWING PROBES**

- Quality
- Availability/Supply - How does your health center manage the SPAQ stock? Where do you receive supply from? How often do you receive supply etc.?
- Price/Cost
- Effectiveness
- Any other aspect?

39. Thinking about the most recent SMC campaign that your health center conducted, how many CHW were involved?

40. Would you say that this is the ideal number of CHWs needed to run a successful SMC campaign?

    If no: In your opinion, what is the ideal number of CHWs involved in an SMC campaign? Why do you say this?

    If yes: Why do you say this?

41. Who is in charge of the CHWs for SMC?

42. How often do the CHWs receive training?

43. How does your health center manage the SPAQ stock?

44. How do the CHWs get the SPAQ supply?

45. How regularly do CHWs come to replenish their stocks?

46. Thinking of the last SMC campaign did your health center experience out of stock?

    If yes:
    - What was the reason for running out of stock?
    - For how long did your center not have any stock?
    - Approximately, how many children missed their dose?

    If no:
    - Did you experience out of stock during any of the past SMC campaigns?

      If yes:
      - What was the reason for running out of stock?
      - For how long did your center not have any stock?
      - Approximately, how many children missed their dose?

    If the SMC campaign is still running: Do you expect an out of stock situation during the current SMC campaign?

47. In an average month of the last SMC campaign, out of all the children that visited your health center what % of children received SPAQ?

If not all received: Please tell me the reasons why not all children that visited your health center receive SPAQ.

48. What % of all the children living in the catchment area of your health center do you estimate did not receive SPAQ during the last SMC campaign?

49. In your opinion what were the reasons why these children did not receive SMC (for example: children not at home because they have to help their parents in field, are away visiting family, parents do not want to give medication to a healthy child etc.).

50. How frequently do you estimate children miss the 2nd and 3rd doses of SMC?

51. Do you have any measures in place that make sure that children receive the 2nd and 3rd doses?

    If yes: What are these measures?

<u>Intermittent preventive treatment for infants (IPTi):</u>

52. Have you heard about an intervention called IPTi (Intermittent preventive treatment for infants)?

    If yes:
    - Please describe what you know about IPTi?
    - Do you think it would be useful for your community?
    - What benefits do you see in IPTi?
    - What disadvantages do you see in IPTi?

53. In your view, do you think there are “false beliefs” that are common in the community regarding malaria chemoprevention?

    If yes: Please tell me what these false beliefs are.

Thank you very much for your answers
