# Supplementary material for: Perception of Malaria Chemoprevention Interventions in Infants and Children in Eight Sub-Saharan African Countries: An End User Perspective Study
Source: Trop Med Infect Dis. 2021 May 11;6(2):75. doi: 10.3390/tropicalmed6020075 (PMC8163176; doi:10.3390/tropicalmed6020075)
Supplement: Supplementary file 1 [file tropicalmed-06-00075-s001.zip › tropicalmed-1197343-suppl-for proof checked.pdf]

Tropical Medicine and Infectious Disease

MDPI

Supplementary Material

Table S1. Sample composition Health Center Managers.

	Cameroon	DRC	Ghana	Nigeria	Senegal	Sierra Leone	Tanzania	Uganda	Total
	n = 8	n = 16	n = 8	n = 13	n = 8	n = 15	n = 15	n = 11	n = 94
Practice setting									
Public	100%	44%	100%	100%	50%	93%	67%	55%	79%
Private	0%	56%	0%	0%	50%	7%	33%	45%	21%
Specialty									
Nurse/midwife	75%	6%	50%	23%	100%	33%	20%	91%	39%
Physician/BSc	13%	94%	50%	69%	0%	0%	80%	9%	38%
CHW/CHEW	0%	0%	0%	0%	0%	67%	0%	0%	20%
NA	13%	0%	0%	8%	0%	0%	0%	0%	2%
Professional experience									
Average of years in position	7	8	4	5	5	3	3	5	5
Average of years of experience	16	13	12	21	12	12	6	12	13

Table S2. Sample composition Community Health Workers.

	Cameroon	Ghana	Nigeria	Senegal	Total
	n = 10	n = 10	n = 16	n = 10	n = 46
Professional experience					
Average number of years as CHWs	8.8	6.0	5.0	11.6	7.6
Catchment area					
Average number of children under 5 years of age	660.3	265.4	1174.5	2750.0	976.2
% of household with 0-5y that also have 5-10y children	81%	58%	65%	NA	68%

Table S3. Sample composition Parents.

	Cameroon	Ghana	Nigeria	Senegal	Sierra Leone	Total
	n = 5	n = 5	n = 5	n = 5	n = 5	n = 25
Parent's relationship with child						
Mother	4	4	4	3	4	19
Father		1				1
Mother and Father			1		1	2
Grand Mother				1		1
Grand Father				1		1
Unspecified	1					1
Family composition						
Average number of children	3.6	3	4.6	3.4	2	3.3

Number of children eligible for SMC at the time of the survey	8	8	9	7	NA	32
Number of children eligible for IPTi at the time of the survey	NA	NA	NA	NA	5	5
